# Progression of cardiovascular risk factors in black Africans: 3 year follow up of the SABPA cohort study

**DOI:** 10.1016/j.atherosclerosis.2014.11.019

**Published:** 2015-01

**Authors:** Mark Hamer, Roland von Känel, Manja Reimann, Nico T. Malan, Alta E. Schutte, Hugo W. Huisman, Leone Malan

**Affiliations:** a Department of Epidemiology and Public Health, University College London, London, UK; b Hypertension in Africa Research Team, School for Physiology, Nutrition and Consumer Sciences, North-West University, Potchefstroom, South Africa; c Department of Neurology, Inselspital, Bern University Hospital and University of Bern, Switzerland; d Autonomic and Neuroendocrinological Laboratory Dresden, Department of Neurology, University Hospital Carl Gustav Carus, Dresden University of Technology, Dresden, Germany

**Keywords:** Black African, Biomarkers, Cardiovascular disease, Epidemiology

## Abstract

Recent work identified a high prevalence of modifiable risk factors for cardiovascular disease (CVD) among urban black South Africans. The aim was to track the progression of CVD risk factors in a multi-ethnic sample of South Africans. Participants were 173 black (aged 47.5 ± 7.8 yrs) and 186 white teachers (aged 49.6 ± 9.9 yrs) that were examined at baseline and 3 years follow-up. Blacks demonstrated a substantially higher prevalence of composite CVD burden (defined as history of physician diagnosed heart disease, use of anti-hypertensives, anti-diabetic, or statin medications at either time point) compared to whites (49.1 vs. 32.0%, *p* = 0.012) respectively. After controlling for baseline, the black participants demonstrated greater increases in 24 h systolic and diastolic blood pressure, total cholesterol, fasting glucose, fibrinogen, D-dimer, and waist circumference in comparison with whites. In summary, an adverse progression of CVD risk factors was observed in the whole sample, although to a larger degree in black participants. Aggressive treatment strategies for controlling risk factors in black Africans are needed to reduce the increasing burden of CVD in South Africa.

## 1 Introduction

Recent work identified a high prevalence of modifiable risk factors for cardiovascular disease (CVD) among urban black South Africans [1], [2]. Most estimates of CVD risk factor prevalence in South Africa have been based on data collection at one point in time, although reliable information about trajectories can help to assess the implications of rising prevalence of CVD risk factors on population health, set policy priorities, and evaluate their success.

Existing data on global trends between 1980 and 2008 has shown increases in various CVD risk factors such as body mass index, cholesterol and blood pressure [3], [4], [5], although substantial differences across regions and sexes have been noted. At present little longitudinal data exist on CVD risk factors in multi-ethnic samples from South Africa.

The aim of the Sympathetic Activity and Ambulatory Blood Pressure in Africans (SABPA) study was to create a unique, highly phenotyped cohort of Africans in a well-controlled research setting enabling us to track the progression of CVD risk factors [6]. Here we present data from the 3-year follow up.

## 2 Methods

The study sample comprised urban black and white teachers from the North West Province, South Africa [6]. All teachers (*N* = 2170), enrolled in the 43 schools of the Dr Kenneth Kaunda Education District (Klerksdorp and Potchefstroom), North-West Province South Africa, were invited to participate. Exclusion criteria included pregnancy or lactating women, individuals who donated blood or had been vaccinated in the 3-months prior the commencement of testing, as well as those with a tympanum temperature greater than 37.5 °C. At baseline, (2008/9) 409 teachers were recruited of which 359 attended the 3-year follow-up. All participants provided written informed consent and the study was approved by The Ethics Review Board of the North-West University (Potchefstroom Campus).

The clinical assessment procedures have been described in detail elsewhere [6]. On the morning prior to the clinical assessment, 24-h ambulatory blood pressure monitoring (ABPM) was conducted during the working day (Meditech CE120 CardioTens; Meditech, Budapest, Hungary) on the non-dominant arm. All participants attended the Metabolic Unit Research Facility of the North-West University and were assessed in the morning after an overnight stay. Basic anthropometric data, including height, body mass and waist circumference, and fasting blood samples were collected. Carotid intima media thickness (CIMT) was assessed using a SonoSite Micromaxx ultrasound system (SonoSite, Bothell, WA) and a 6–13 MHz linear array transducer. The ABPM data was analysed using the CardioVisions 1.15 Personal Edition software (Meditech).

Plasma and serum from fasting blood samples were analysed using two sequential multiple analyzers (Konelab 20i; Thermo Scientific, Vantaa, Finland; Unicel DXC 800 - Beckman and Coulter^®^, Germany), enzyme-linked immunosorbent assays (Quantikine Enzyme linked immunosorbent assay, R&D Systems, Minneapolis, MN USA), and viscosity-based clotting method (Immuno-turbimetric method 540 nm, STA Compact, STAGO Diagnostic). Biochemical risk factors included endothelin-1, total and high density lipoprotein (HDL), cholesterol, triglycerides, glycated haemoglobin, glucose, insulin, fibrinogen, D-dimer, interleukin-6, and tumour necrosis factor-α. The intra- and inter-coefficients of variation for all assays were below 10%.

The distribution of all variables was tested for normality and non-normally distributed variables were appropriately transformed prior analyses. We used *χ*^2^ tests to examine differences in prevalence of medication use and history of CVD between blacks and whites. General linear models were used to examine the difference in risk factors between black and white participants at follow-up after controlling for baseline levels of the respective risk factor. In addition, models were adjusted for age, sex, baseline serum cotinine as an objective indicator of smoking, baseline physical activity energy expenditure (24 h Actical accelerometer), history of CVD, and baseline medication (anti-hypertensives, statins, antidiabetics). These co-variables were chosen *a priori* based on associations with the exposure and the independent variables [7]. All analyses were conducted using SPSS (version 20), and statistical significance was set at *p* < 0.05.

## 3 Results

At follow-up, 173 black (aged 47.5 ± 7.8 yrs) and 186 white participants (aged 49.6 ± 9.9 yrs) attended the clinic (representing 86.5% and 89.1% of initial baseline sample, respectively). Reasons for non-attendance at follow up included death (*n* = 9), pregnancy (*n* = 1), lactating (*n* = 1), withdrawal from study (*n* = 39). Participants that withdrew (54% blacks) had similar prevalence of CVD at baseline (14.0 vs. 10.3%, *p* = 0.43), and did not differ in their reported use of antihypertensive medication (16.0 vs. 24.5%, *p* = 0.18) compared to participants attending follow-up. They also did not differ in relation to other risk factors such as total: HDL cholesterol ratio (4.55 ± 1.94 vs. 4.77 ± 1.84, *p* = 0.44) and glucose (5.32 ± 1.24 vs. 5.70 ± 1.58 mmol/l, *p* = 0.11).

At baseline, physician diagnosed heart disease was prevalent in 9.0% and 11.4% of black and white participants respectively. Anti-hypertensive medication was used in 36.6% of black participants and 66.0% met criteria for hypertension based on 24hr ABPM (>130/80 mmHg) [8] alone; in comparison only 13.5% of white participants used anti-hypertensives and 39.2% were classified as hypertensive based on ABPM alone. Statins were used in 1.0% and 4.9% of black and whites, respectively, whereas anti-diabetic medication was used in 5.0% and 1.0% respectively. At follow-up, there was no difference in incident use (new users that did not report being medicated at baseline) of anti-hypertensives between black and white participants (9.8% vs. 11.8%, *p* = 0.72); incident use of statins was lower in blacks compared to whites (1.7% vs. 13.4%, *p* = 0.001); incident use of anti-diabetic medication was greater in blacks compared with whites (10.4% vs. 4.8%, *p* = 0.03). We calculated the accumulative prevalence of CVD burden, based on a composite score comprising history of physician diagnosed heart disease, use of anti-hypertensives, anti-diabetic, or statin medications at either time point. Blacks demonstrated a substantially higher prevalence of composite CVD burden compared to whites (49.1 vs. 32.0%, *p* = 0.012).

The mean averages and standard errors for CVD risk factors at baseline and follow up in blacks and whites are presented in Table S1. After controlling for baseline, the black participants demonstrated greater increases in 24 h systolic and diastolic blood pressure, total cholesterol, glucose, fibrinogen, D-dimer, and waist circumference in comparison with whites (see Table 1). In contrast, whites demonstrated a greater increase in CIMT, cross sectional wall area and tumour necrosis factor-α. Nevertheless, absolute levels of tumour necrosis factor-α remained higher in blacks at baseline and follow-up (Table S1).

## 4 Discussion

The aim of this study was to examine the progression of CVD risk factors in a unique, highly phenotyped cohort of Africans. Previous work has identified a high prevalence of modifiable risk factors among urban black Africans [1], [2], although we are not aware of any longitudinal study that has examined trajectories in risk factors in this population. Thus, this is one of the first studies to demonstrate overall an adverse progression of risk factors over 3 years follow-up in black South Africans compared with whites. The black participants also demonstrated a different pattern of medication use, reporting greater use of anti-hypertensives and anti-diabetics, but lower statin use than whites.

The current acculturation process in black Africans is characterized by detrimental behavioural lifestyle factors such as unhealthy dietary habits, sedentary behaviour, excess alcohol intake and tobacco use that might partly explain the dramatic increase in CVD. Differences in lifestyle risk factors between black and white SABPA participants have been demonstrated at baseline [7], and other work suggested smoking and waist circumference were predictive of incident hypertension over 5 years follow up in black Africans [9]. Although South Africa has seen an overall trend for reduced smoking rates over the last 20 years, there is marked heterogeneity. For example, lower socio-economic groups have demonstrated an increasing trend for use of roll-your-own cigarettes [10]. The present analyses were adjusted for objective measures of smoking and physical activity, thus adverse CVD risk might also be explained by other variables, such as chronic stress exposure [11].

Interestingly, white participants demonstrated greater increases in CIMT than blacks despite higher reported usage of statin medication and lower total cholesterol levels. Albeit, similar prevalence of CIMT stenosis was observed in whites (26.0%) compared to blacks (24.9%). This is partly consistent with previous findings where black Africans were more likely to be diagnosed with symptomatic cerebrovascular disease (hypertension, ischaemic heart disease and diabetes) [12] consistent with the risk factors observed in the present sample of black Africans.

Ethnic differences in coagulation are still poorly understood with studies showing enhanced clotting but also increased bleeding tendency in various clinical settings in Africans [13]. We found greater increases in fibrinogen and D-dimer over follow-up in blacks than whites, and this potential predisposition to low-grade hypercoagulability might contribute to higher risk of atherothrombotic CVD, including stroke.

The limitations of this study should be highlighted. Firstly, the sample consisted of urban-dwelling teachers that may not be representative of the South African population. Data on medication and diagnosis of prevalent CVD were based on self report that might have introduced bias.

In summary, there appears to be a worsening CVD risk profile in both black and white participants over a 3-year period, although there is a difference in the distribution of these markers. Overall, there was a greater worsening/progression of CVD risk factors in black compared to white participants. Aggressive treatment strategies for controlling CVD risk factors in black Africans are needed to reduce the increasing burden of CVD in South Africa.

## Conflicts of interest

None declared.

## Funding

The study was partly funded by The Metabolic Syndrome Institute, France; the Medical Research Council, National Research Foundation, North-West University, and North-West Department of Education, South Africa. MH is supported by the British Heart Foundation (RE/10/005/28296). The funders played no role in the design and conduct of the study, in the collection, analysis, and interpretation of the data, and in the preparation, review, or approval of the manuscript.

## Figures and Tables

**Table 1:** Comparison between white (reference group) and black participants of CVD risk factor progression over a three-year follow-up period.

Risk factor	Adjusted coefficient (95% CI)	*p*-value
24 h systolic blood pressure	6.02 (3.51, 8.55)	<0.001
24 h diastolic blood pressure	3.57 (1.96, 5.17)	<0.001
CIMT	−0.048 (−0.033, −0.064)	<0.001
Cross sectional wall area	−1.27 (−0.73, −1.81)	<0.001
Endothelin-1	0.21 (−0.99, 1.41)	0.73
HDL cholesterol	−0.02 (−0.08, 0.04)	0.50
Total cholesterol	0.65 (0.45, 0.86)	<0.001
Total: HDL cholesterol ratio	0.86 (0.64, 1.08)	<0.001
Triglycerides	0.26 (−0.11,0.17)	0.72
Glycated haemoglobin	0.13 (−0.05, 0.30)	0.16
Glucose	1.22 (0.91, 1.52)	<0.001
Insulin	0.35 (−0.94, 1.65)	0.59
Fibrinogen	0.55 (0.41, 0.69)	<0.001
D-dimer	132.15 (31.36, 232.94)	0.01
Interleukin-6	−1.48 (−4.37, 1.42)	0.32
Tumour necrosis factor-α	−0.57 (−1.00, −0.14)	0.01
BMI	−0.24 (−0.73, 0.25)	0.33
Waist	1.92 (0.24, 3.59)	0.03

Coefficients reflect differences between white (ref) and black Africans, adjusted for age, sex, serum cotinine, physical activity energy expenditure, history of CVD, medication, and baseline levels of the respective risk factor. Where: CIMT, carotid intima media thickness; HDL, high density lipoprotein; BMI, body mass index.
