# Supplementary material for: Progression of cardiovascular risk factors in black Africans: 3 year follow up of the SABPA cohort study
Source: Atherosclerosis. 2015 Jan;238(1):52–4. doi: 10.1016/j.atherosclerosis.2014.11.019 (PMC4726657; doi:10.1016/j.atherosclerosis.2014.11.019)
Supplement: Supplementary file 1 [file mmc1.pdf]

SUPPLEMENTARY DATA

Table S1. Descriptive data on CVD risk factors at baseline and follow up in the SABPA cohort study (2008-2011).

**Risk factor**	**Whites** (n=186)	**Blacks** (n=173)
24hr Systolic BP (mmHg)		
Baseline	124.5±1.0	133.1±1.0
Follow-up	123.2±1.0	135.8±1.1
24hr Diastolic BP (mmHg)		
Baseline	77.2±0.6	83.7±0.7
Follow-up	75.6±0.6	83.9±0.7
Carotid Intima media thickness (mm)		
Baseline	0.65 ± 0.01	0.68 ± 0.01
Follow-up	0.70 ± 0.01	0.68 ± 0.01
Carotid cross-sectional wall area (mm^2)		
Baseline	13.3 ± 0.27	13.9 ± 0.28
Follow-up	15.1 ± 0.24	14.2 ± 0.25
Endothelin-1 (pg/mL)		
Baseline	3.15 ± 0.50	2.72 ± 0.51
Follow-up	2.11 ± 0.39	2.62 ± 0.42
Total: HDL Cholesterol ratio		
Baseline	5.08±0.12	4.43±0.13
Follow-up	4.45±0.11	4.90±0.11
Triglycerides (mmol/l)		
Baseline	1.24±0.07	1.44±0.07
Follow-up	1.19±0.06	1.36±0.06
Glycated haemoglobin (%)		
Baseline	5.52±0.07	6.11±0.07
Follow-up	5.58±0.08	6.23±0.08
Glucose (mmol/l)		
Baseline	5.70±0.11	5.71±0.12
Follow-up	4.42±0.13	5.72±0.13
Insulin (uU/mL)		
Baseline	12.31 ± 0.68	14.87 ± 0.71
Follow-up	10.38 ± 0.55	11.83 ± 0.58
Fibrinogen (g/L)		
Baseline	3.07±0.05	5.54±0.05
Follow-up	3.82±0.06	5.72±0.06
D-dimer (µg/L)		
Baseline	342.87 ± 30.02	471.24 ± 31.69
Follow-up	326.00 ± 35.46	506.60 ± 37.44
Interleukin-6 (pg/mL)		
Baseline	1.16 ± 0.40	2.07 ± 0.42
Follow-up	3.70 ± 1.03	3.05 ± 1.09
Tumor necrosis factor-α (pg/mL)		
Baseline	1.80 ± 0.19	3.31 ± 0.20
Follow-up	2.73 ± 0.14	3.05 ± 0.15
Body mass index (kg/m^2)		

Baseline	27.72 ± 0.46	30.04 ± 0.48
Follow-up	28.75 ± 0.48	30.75 ± 0.49
Waist circumference (cm)		
Baseline	93.49 ± 1.08	93.84 ± 1.12
Follow-up	96.14 ± 1.12	98.19 ± 1.16

Data are mean±SEM, adjusted for age and sex.
